# Evaluación *in vitro* de la patogenicidad de los hongos aislados en la región de Urabá (Antioquia, Colombia) contra larvas de *Aedes aegypti*

**DOI:** 10.7705/biomedica.7520

**Published:** 2024-11-06

**Authors:** Dairon Andrés Machado-Agudelo, María Alejandra García, Manuel E. Rueda-Páramo, Nadya Lorena Cardona

**Affiliations:** 1 Grupo de Fitopatología y Biotecnología de Hongos - FITOBIOL, Instituto de Biología, Facultad de Ciencias Exactas y Naturales, Universidad de Antioquia, Medellín, Colombia Universidad de Antioquia Facultad de Ciencias Exactas y Naturales Universidad de Antioquia Medellín Colombia; 2 Centro de Estudios Parasitológicos y de Vectores - CEPAVE, Consejo Nacional de Investigaciones Científicas y Técnicas - CONICET, Universidad Nacional de La Plata - UNLP, La Plata, Argentina Universidad Nacional de La Plata Consejo Nacional de Investigaciones Científicas y Técnicas - CONICET Universidad Nacional de La Plata La Plata Argentina

**Keywords:** Aedes, mosquitos vectores, control de mosquitos, agentes de control biológico, Trichoderma, dengue, Aedes, mosquito vectors, mosquito control, biological control agents, Trichoderma, dengue

## Abstract

**Introducción.:**

*Aedes aegypti* es un vector importante de enfermedades arbovirales como el dengue, entre otras. Las estrategias tradicionales de control, como el uso de insecticidas, han perdido eficacia debido a la aparición de resistencia en las poblaciones de mosquitos. El control biológico y el uso de hongos biocontroladores se presentan como alternativas viables y amigables con el medio ambiente.

**Objetivo.:**

Evaluar la patogenicidad *in vitro* de aislamientos del género *Trichoderma* -obtenidos del Urabá antioqueño- sobre larvas de *Ae. aegypti,* y determinar la concentración letal media (CL_50_) y el tiempo letal medio (TL_50_) del más patógeno.

**Materiales y métodos.:**

Mediante el método de larvas centinelas con especímenes de *Ae. aegypti,* se logró aislar hongos de cuerpos de agua de la región del Urabá (Antioquia). Los aislamientos se caracterizaron a nivel morfológico y molecular, para determinar su identidad taxonómica. Se llevaron a cabo pruebas de patogenicidad *in vitro* utilizando larvas de *Ae. aegypti* en los estadios L_2_ y L_3_. Posteriormente, se seleccionó una cepa para determinar su concentración letal media y tiempo letal medio.

**Resultados.:**

La cepa AP-91 de *Trichoderma* sp. causó porcentajes altos de mortalidad en poblaciones de larvas de *Ae. aegypti.* Se obtuvo una concentración letal media de 1,8 * 10^7^ conidios/ml y un tiempo letal medio de 20,67 horas.

**Conclusión.:**

La cepa AP-91 tiene potencial para el control biológico de *Ae. aegypti* y puede ser una candidata idónea para usar en el manejo integrado de vectores mediante su cultivo escalado. La investigación sugiere explorar los compuestos y enzimas producidos por esta cepa para comprender mejor su patogenicidad.

## Abstract (en)

**Introduction.:**

*Aedes aegypti* is an important vector of arboviral diseases like dengue among others. Traditional control strategies, such as the use of insecticides, have lost effectiveness due to the emergence of resistance in mosquito populations. Biological control and fungi applied for biocontrol are presented as viable and ecological alternatives.

**Objective.:**

To evaluate *in vitro* pathogenicity of *Trichoderma* sp. isolates obtained from Urabá (Antioquia) on larvae of *Ae. aegypti,* and to determine the mean lethal concentration and mean lethal time of the most pathogenic isolate.

**Materials and methods.:**

Using the sentinel larvae method of *Ae. aegypti,* fungi were isolated from water bodies in the Urabá region (Antioquia). The isolates were characterized morphologically and molecularly to determine their taxonomic identity. Pathogenicity tests were performed *in vitro* on *Ae. aegypti* larvae in the L_2_/L_3_ stages. Subsequently, a strain was selected to establish its mean lethal concentration and mean lethal time.

**Results.:**

*Trichoderma* sp. strain AP-91 caused high mortality in larval populations of *Aedes aegypti.* We estimated a mean lethal concentration of 1.8 * 10^7^ conidia/ml and a mean lethal time of 20.67 hours.

**Conclusion.:**

The strain AP-91 showed potential for its use as biological control of *Ae. aegypti,* making it a suitable candidate for scale-up cultures applied to integrated vector management. This research suggests exploring compounds and enzymes produced by the AP-91 strain to understand better its pathogenicity.

*Aedes (Stegomyia) aegypti* (Linnaeus, 1762) (Diptera: Culicidae) es un insecto hematófago, antropofílico, actualmente cosmopolita, que llegó desde África a las Américas hace 350 años y que posee una gran capacidad de adaptación a diversos ambientes ^1^. Esta especie representa un importante factor de riesgo para la salud pública, debido a su capacidad como vector de diferentes arbovirus que causan enfermedades como el dengue, el Zika, la fiebre del chikunguña y la fiebre amarilla ^1^. En Colombia, entre el 2010 y el 2016, el 40,8 % de los 5360.134 casos reportados de enfermedades transmitidas por vectores corresponden a aquellas transmitidas por *Ae. aegypti,* con una especial prevalencia en áreas urbanas ^2^.

La principal estrategia para el control de *Ae. aegypti* ha sido la reducción de la población de mosquitos ^3^, y los métodos más frecuentemente utilizados han sido la eliminación de criaderos y el uso de insecticidas, como piretroides, carbamatos y organofosforados ^4^. Sin embargo, el uso intensivo de plaguicidas químicos está generando resistencia en las poblaciones de *Ae. aegypti* a nivel mundial ^4^ y en el territorio colombiano ^5^^-^^7^

Una alternativa interesante para el manejo de las poblaciones de *Ae. aegypti* es el control biológico ^8^. Se han estudiado diversos métodos, como el uso de enemigos naturales: peces, copépodos y larvas depredadoras, bacterias, hongos y oomicetos (Oomycetes) ^8^^,^^9^. Los hongos biocontroladores se presentan como una opción interesante. Estos hongos, ampliamente estudiados en el sector agrícola, son enemigos naturales del mosquito y desempeñan un papel importante en los ecosistemas tropicales ^8^^,^^10^^,^^11^.

Los hongos biocontroladores tienen la capacidad de penetrar la cutícula de los insectos o ingresar por aberturas naturales, provocando su muerte mediante diversos mecanismos ^12^^,^^13^. Un estudio realizado en 2021 ^14^ con el hongo *Metarhizium anisopliae,* cepa MET-GRA4, aplicado en Malasia sobre larvas de *Ae. aegypti,* produjo una mortalidad del 100 %. Además, una investigación reciente ^15^ demostró la actividad biolarvicida de una cepa de *Trichoderma* spp. contra el tercer estadio (L_3_) *de Ae. aegypti,* evidenciando el potencial de este género fúngico como agente patógeno de insectos.

En la presente investigación se evaluó la patogenicidad *in vitro* de dos cepas de *Trichoderma* spp., obtenidas en cuerpos de agua de la región del Urabá antioqueño, contra larvas (L_2_-L_3_) de *Ae. aegypti.* Asimismo, se determinaron la concentración letal media (CL_50_) y el tiempo letal medio (TL_50_) para el aislamiento más patógeno.

## Materiales y métodos

### Procedencia de los estadios larvales de Aedes aegypti

Los huevos de *Ae. aegypti* fueron provistos por una colonia de cría mantenida en el Laboratorio de Entomología Médica de la Universidad de Antioquia. Para la obtención de las larvas en los estadios de desarrollo seleccionados para las pruebas, se introdujeron los huevos en contenedores con 700 ml de agua destilada estéril y se incubaron a temperatura ambiente (25 - 28 °C). Las larvas recibieron alimento para peces, previamente esterilizado, hasta alcanzar el estadio L_2_-L_3_.

### Obtención de los aislamientos fúngicos

Se realizaron salidas de campo en los municipios de Carepa y Apartadó, y en el distrito de Turbo del departamento de Antioquia (Colombia). Se buscaron cuerpos de agua en áreas periurbanas y rurales con el propósito de aislar hongos asociados con estadios larvales de *Ae. aegypti.*

Para ello, se aplicó la metodología de «larvas centinelas» ^16^, que consistió en el uso de larvas de *Ae. aegypti* ubicadas en trampas expuestas a los microorganismos presentes naturalmente en los cuerpos de agua, sin permitir su liberación al entorno. Estas trampas se recuperaron después de 48 horas de exposición.

Se midieron variables abióticas en los cuerpos de agua muestreados, las cuales serán discutidas en una publicación posterior. Las larvas centinelas fueron sometidas a lavados sucesivos en agua destilada estéril durante un minuto, luego con alcohol al 70 % durante 30 s, agua destilada estéril durante un minuto, hipoclorito de sodio al 2 % durante 30 s y agua destilada estéril durante un minuto.

A partir de las muestras recolectadas y desinfectadas, se realizaron aislamientos fúngicos sembrando las larvas centinelas en medio agar Sabouraud modificado con extracto de levadura (SDAY), compuesto por 1,2 g/L de peptona, 3 g/L de dextrosa, 1,2 g/L de extracto de levadura y 15 g/L de agar bacteriológico ^17^. A este medio se añadieron 0,5 ppm de cloranfenicol, 0,1 ppm de ampicilina y 0,05 ppm de gentamicina. Las muestras se incubaron a 25 °C durante 72 horas.

Los aislamientos obtenidos se conservaron en el Laboratorio de Fitopatología y Biotecnología de Hongos - FITOBIOL - de la Universidad de Antioquia. La relación entre los aislamientos obtenidos y las variables abióticas, así como el impacto de estas en los procesos de bioprospección con larvas centinelas, serán tratados con mayor detalle en una publicación posterior.

Las dos cepas utilizadas en este estudio, identificadas como AP-61 y AP-91, fueron aisladas de dos humedales presentes en una finca ganadera en la zona rural del municipio de Apartadó, a 200 m de la troncal del Urabá, rodeados de árboles de alto porte y gramíneas (AP-61: 7° 54' 0,3'' norte, 76° 37' 40,2'' oeste; 28 m.s.n.m.; AP-91: 7° 54' 59,8'' norte, 76° 37' 41,8'' oeste; 21 m.s.n.m.).

### Caracterización morfológica y molecular de las cepas

Para la identificación morfológica, las cepas se sembraron en agar papa dextrosa y se incubaron durante 7 a 15 días a 25 °C. Se tiñeron con azul de lactofenol y se analizaron con microscopía óptica; se describieron las estructuras reproductivas y se utilizaron claves taxonómicas ^18^^,^^19^ para su clasificación a nivel de género.

Para la extracción del ADN, requerida para los análisis moleculares, se produjo biomasa de las cepas fúngicas en medio líquido Sabouraud dextrosa (Merck^®^) con 10 g/L de extracto de levadura (SDYb) (Scharlau^®^). Durante tres días, las cepas se sometieron a agitación orbital (agitador Mazzine, Indulab^®^) a 0,145g a 25 °C. Después, la biomasa se filtró y se sometió a maceración con nitrógeno líquido. La muestra resultante se procesó según el protocolo de extracción de ADN publicado por Miranda y Sandoval ^20^.

Se amplificó la región ITS (ITS1, ITS2 y 5,8S ARNr) usando los cebadores ITS4 (5'-TCCTCCGCTTATTGATATGC-3') e ITS5 (5'-GAAGTAAAAGTCGTAACAAGG-3') ^21^. La amplificación se llevó a cabo en un termociclador de marca Applied Biosystems MiniAmp con 25 μl de volumen total de reacción por muestra, que contenía 2,5 μl de solución tampón 10X (Smobio^®^), 2 mM de MgCl_2_ (Smobio^®^), 0,2 mM de dNTPs (Smobio^®^), 0,2 pmol/μl de cada cebador (AccuOligo^®^, Bioneer), 0,06 U/μl de polimerasa Taq (Smobio^®^) y 20 ng/μl de ADN.

El ciclo de amplificación comenzó con una desnaturalización inicial a 95 °C durante 5 minutos, seguida de 35 ciclos de desnaturalización a 95 °C durante 1 minuto, alineación a 55 °C durante 1 minuto y extensión a 72 °C durante 1,5 minuto. Finalmente, se hizo una extensión final a 72 °C durante 10 minutos. Los productos amplificados en la PCR se visualizaron en un gel de agarosa (1 % p/v) en solución tampón con 0,5 X TAE (tris, acetato, EDTA) a 80 V durante 40 minutos. Los fragmentos amplificados se enviaron a la Unidad de Servicios de Secuenciación "Gabriel de Jesús Bedoya Berrío" de la Universidad de Antioquia.

Las secuencias obtenidas se revisaron y editaron mediante el programa FinchTV, y se compararon con las bases de datos de dominio público del NCBI *(National Center for Biotechnology Information)* y de UNITE ^22^, mediante la herramienta BLASTN para alinear secuencias de nucleótidos.

### Pruebas de patogenicidad contra larvas de Aedes aegypti

Se emplearon conidios de las cepas de *Trichoderma* AP-61 y AP-91 en las pruebas de patogenicidad. Para alcanzar el volumen requerido, se realizó la producción de conidios en arroz como sustrato nutritivo. Para el inóculo, se usaron los cultivos mantenidos en cajas de Petri de 100 mm con agar papa dextrosa.

Los conidios obtenidos después de 15 días de incubación se rasparon superficialmente y se homogenizaron en 10 ml de agua destilada estéril con 0,01 % de Tween 80 por cada caja de Petri. Posteriormente, se tomaron alícuotas de 5 ml para inocular botellas que contenían 50 g de arroz esterilizado y 30 ml de agua destilada estéril con 2 % de ácido láctico. Estas botellas se incubaron durante 15 días a 25 °C, bajo iluminación con luz blanca hasta producir conidios.

Se añadieron entre 100 y 120 ml de agua destilada estéril con 0,01 % de Tween 80 a cada botella que contenía al hongo crecido en arroz. Los conidios se desprendieron con espátulas estériles y cada preparación se filtró con gasa estéril, trasvasándola a un envase de vidrio de 150 ml previamente esterilizado. Se hicieron alícuotas de la preparación y, luego, diluciones seriadas hasta alcanzar el orden de magnitud de 10^-3^. Las concentraciones se determinaron mediante conteo en cámara de Neubauer y la fórmula presentada por Vélez *et al.*^23^.

Los bioensayos se llevaron a cabo en el laboratorio de FITOBIOL en condiciones estándar de humedad y temperatura. La unidad experimental consistió en un vaso plástico de 207 ml (TAM^®^ Colombia) con un volumen final de 50 ml de agua destilada estéril con 0,01 % de Tween 80, más el inóculo a una concentración de 10^7^ conidios/ml.

En cada vaso se adicionaron 20 larvas L_2_/L_3_ de *Ae. aegypti.* Estos vasos se cubrieron con velo suizo y cintas elásticas. Durante el experimento, las larvas se alimentaron con dos o tres granos diarios previamente esterilizados de concentrado para peces ornamentales ^24^. El control negativo consistió en 50 ml de agua destilada estéril con 0,01 % de Tween 80 en ausencia de conidios.

Cada tratamiento se replicó cinco veces y la mortalidad de cada unidad experimental se registró a las 24, 48 y 72 horas. Se consideró que una larva estaba muerta si no presentaba ningún tipo de movilidad después de la estimulación con pincel de punta fina o pipeta Pasteur.

### Determinación de la concentración letal 50

Con el objetivo de determinar la CL_50_ de las larvas de *Ae. aegypti* cultivadas con la cepa AP-91, se prepararon cinco concentraciones de conidios por mililitro: 5 * 10^6^, 1 * 10^7^, 4 * 10^7^, 8 * 10^7^ y 1 * 10^8^. Las unidades experimentales de 20 larvas cada una y el grupo control fueron tratados como se describió en el experimento anterior. Cada tratamiento se replicó cuatro veces con dos repeticiones en tiempos diferentes. La mortalidad de las larvas fue registrada a las 72 horas.

### Determinación del tiempo letal 50

Se usó la cepa AP-91 a una concentración de 1 * 10^8^ conidios/ml y, como control, se utilizó agua destilada estéril con 0,01 % de Tween 80. Cada tratamiento contó con cuatro repeticiones y dos réplicas en el tiempo; cada unidad experimental de los diferentes tratamientos tuvo 10 larvas en estadio L_2_-L_3_ en un volumen de 50 ml. Igualmente, se dispuso de un control negativo de larvas sin conidios. La lectura de mortalidad se hizo cada ocho horas, hasta completar las 40 horas.

### Verificación de los agentes causales

Se llevaron a cabo observaciones en el estereoscopio para detectar signos característicos de micosis, como melanización, deformidades morfológicas o el crecimiento del hongo en la larva ^17^. Además, se desinfectaron cuatro larvas muertas por vaso mediante lavados sucesivos con agua destilada estéril durante 1 minuto, 70 % de etanol durante 30 s, agua destilada estéril durante 1 minuto , 2 % de hipoclorito de sodio durante 30 s y agua destilada estéril nuevamente durante 1 minuto . Posteriormente, las larvas se sembraron en medio agar papa dextrosa acidificado para confirmar las causas de mortalidad. Después de seis días de incubación, se tomaron muestras del hongo emergente que luego se observaron al microscopio para verificar morfológicamente que se trataba de *Trichoderma* sp.

### Análisis estadístico

Para todos los bioensayos se siguió un diseño completamente aleatorizado y el porcentaje de mortalidad se tomó como variable de respuesta. Cuando se obtuvo un promedio de mortalidad superior al 5 % en el grupo de control, se corrigieron los porcentajes de mortalidad de los tratamientos ^25^ con la fórmula de Abbot, modificada por Schneider-Orelli (1947) ^17^.

Todos los resultados se sometieron a la prueba de normalidad de residuos de Shapiro-Wilk y a la de homocedasticidad de Levene. Los datos del primer bioensayo se analizaron mediante un análisis de varianza unifactorial (ANOVA), seguido de una prueba *post hoc* de significación honesta *(Honestly Significant Difference,* HSD) de Tukey. Mediante un análisis Probit, se obtuvo la CL_50_ y el TL_50_ con sus correspondientes intervalos de confianza (IC). Todas las pruebas se realizaron con una confianza del 95 %. Las operaciones se llevaron a cabo en el programa R, versión 4.3.1., utilizando los paquetes agricolae ^26^, FSA ^24^, dplyr ^27^, ggplot2 ^28^ y ecotox ^29^.

### Consideraciones éticas

Los aislamientos se realizaron amparados en el permiso marco de recolección de la Universidad de Antioquia (Resolución de la Autoridad Nacional de Licencias Ambientales 1263 del 8 de octubre de 2015). Durante la toma de muestras y el trabajo de laboratorio, se aplicaron las medidas básicas de bioseguridad, de acuerdo con la Resolución 8430 de 1993 del Ministerio de Salud y Protección Social de Colombia, teniendo en cuenta que los microrganismos trabajados están categorizados dentro del grupo de riesgo I.

## Resultados

### Identificación morfológica y molecular

El análisis morfológico macroscópico y microscópico, y la evidencia reportada en la bibliografía consultada, sugieren que las cepas AP-91 y AP-61 pertenecen al género *Trichoderma.* Esta identificación coincide con los resultados obtenidos en el análisis molecular. En este sentido, las secuencias amplificadas a partir de la región ITS ribosómica presentaron un 99 % de similitud con las de *Trichoderma,* publicadas en las bases de datos consultadas.

Estos hallazgos permitieron la identificación de la cepa AP-61 como perteneciente a la especie *T. hamatum,* mientras que no fue posible identificar la especie de AP-91. Para este fin, se tendrían que realizar otros estudios, empleando marcadores moleculares como el factor de elongación de la traducción *(Translation Elongation Factor,* TEF) y el gen de la ß-tubulina ^30^.

### *Pruebas de patogenicidad y determinación de la CL*_*50*_ *y el TL*_*50*_

**Figura 1:**
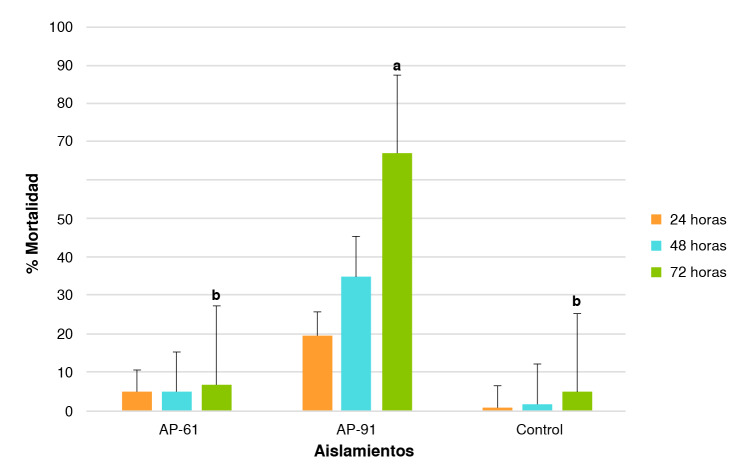
Porcentaje de mortalidad promedio *in vitro* de las cepas AP-61 y AP-91 sobre larvas L_2_/L_3_ de *Aedes aegypti* y su respectivo control, medido a las 24, 48 y 72 horas (las barras negras representan el error estándar). Las letras a y b sobre los tratamientos a las 72 horas, indican diferencias significativas según la prueba *Honestly Significant Difference* (HSD) de Tukey, p < 0,001).

Mediante las pruebas realizadas, se confirmó la patogenicidad de la cepa AP-91 en los estadios larvales de *Ae. aegypti.* La mortalidad generada por la cepa AP-91 se diferenció estadísticamente de las obtenidas con la cepa AP-61 y el control (ANOVA, gl = 2; F = 37,055; p < 0,001). La cepa AP-91 generó el mayor porcentaje de mortalidad durante el periodo de estudio. La mortalidad de las larvas tratadas con esta cepa aumentó con el paso del tiempo, pasando del 20 al 35 y al 67 % a las 24, 48 y 72 horas, respectivamente. Por su parte, la mortalidad obtenida con la cepa AP-61 no superó el 50 % durante las 72 horas de seguimiento y no se diferenció estadísticamente del control (figura 1).

En virtud de los resultados obtenidos, se seleccionó la cepa AP-91 para las pruebas de virulencia. Las larvas tratadas con los conidios de esta cepa, en las concentraciones de 10^8^ y 10^7^ conidios/ml, presentaron mortalidades del 98 y el 80 % después de 72 horas de exposición (cuadro 1). Por otro lado, la CL_50_ fue de 1,8 * 10^7^ conidios/ml (IC_95%_: 1,6 x 10^7^ - 2,1 x 10^7^; pendiente = 2,27 + 0,12). La mortalidad de los controles fue inferior al 20 %, validando la calidad de los datos obtenidos según el umbral establecido por la Organización Mundial de la Salud (20 %) (25). El TL_50_ para la concentración de conidios fue de 20,67 horas (IC_95%_: 18,1 - 23,8; pendiente = 2,27 + 0,28).

**Cuadro 1:** Porcentajes de mortalidad promedio de la cepa AP-91 sobre larvas L_2_/L_3_ de *Aedes aegypti* en cinco concentraciones diferentes para la determinación de la concentración letal 50 (CL_50_) y en cinco tiempos diferentes con una concentración de 1 * 10^8^ conidios/ml para la determinación del tiempo letal 50 (TL_50_)

Tiempo (horas)	Promedio de mortalidad (%)	Concentración (conidios/ml)	Promedio de mortalidad (%)
8	20	5 * 10^6^	13,12
16	35	1 * 10^7^	20,62
24	55	4 * 10^7^	80,62
32	65	8 * 10^7^	88,12
40	77,5	1 * 10^8^	98,12

**Figura 2:**
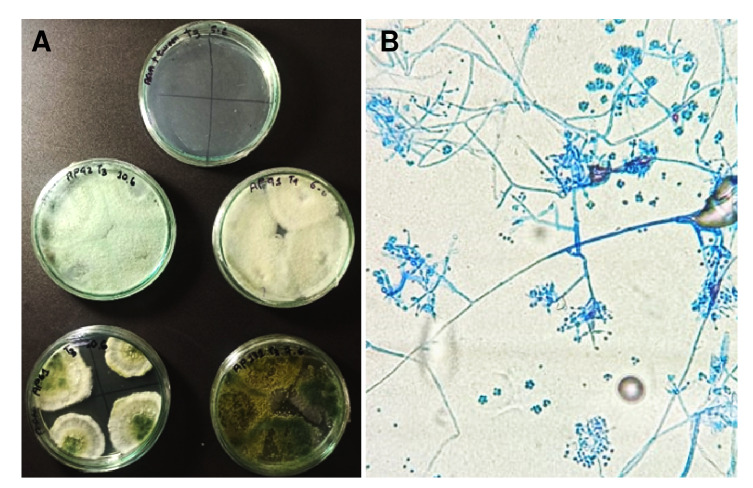
A) Crecimiento de las cepas de *Trichoderma* spp. a partir de las larvas que murieron en los bioensayos de patogenicidad, sembradas en medio de agar papa dextrosa (PDA) acidificado. B) Conidióforos y conidios de *Trichoderma* spp.

La mortalidad de las larvas en respuesta a la exposición del agente patógeno se corroboró en todos los casos mediante observación con microscopio óptico. Durante el análisis se buscaron signos de infección como deformidades y estructuras melanizadas. La presencia del agente patógeno se confirmó mediante su aislamiento en medio de cultivo a partir de las larvas muertas (figuras 2 y 3).

**Figura 3:**
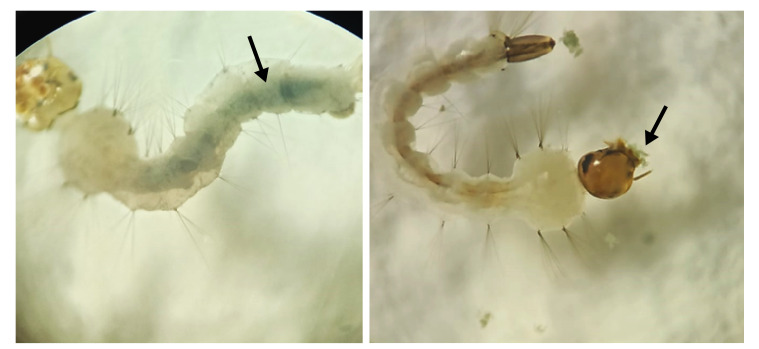
Crecimiento de *Trichoderma* sp. (flecha) dentro del cuerpo de las larvas expuestas a los tratamientos con la cepa AP-91 durante los bioensayos

## Discusión

El método de larvas centinela fue reportado por primera vez en el 2016. En esa ocasión, permitió a los investigadores aislar ocho cepas del oomiceto entomopatógeno *Leptolegnia chapmanii*^16^. Con este estudio, se validó el método del insecto centinela para la prospección de agentes patógenos en la naturaleza. Como resultado, se aislaron dos cepas del hongo *Trichoderma.* La cepa AP-91 presentó patogenicidad y virulencia elevadas en las pruebas practicadas *in vitro.*

La patogenicidad de hongos biocontroladores ha sido reportada por otros autores e incluyen aislamientos de las especies *Gliocladium virens, Beauveria bassiana* y *Metarhizium anisopliae,* y otros organismos como los oomicota.

En dichos estudios, las larvas de *Ae. aegypti* se pusieron en contacto con suspensiones de los microrganismos mencionados, para evaluar su capacidad patógena; asimismo, la patogenicidad del género *Trichoderma* se ha evaluado mediante la aplicación directa de suspensiones de conidios sobre estadios larvarios de diferentes especies de culicidos o por contacto de filtrados de crecimiento en medio de cultivo con quitina o larvas desecadas de *Ae. aegypti.*^15^^,^^16^^,^^31^^-^^38^.

Lo anterior demuestra el potencial de *Trichoderma* sp. para el manejo de diferentes tipos de insectos vectores y abre la posibilidad de realizar futuros estudios con AP-91.

La diferencia evidenciada entre la patogenicidad de las dos cepas nativas (AP-61 y AP91) y otras cepas de referencia, podria estar asociada con diferencias en los mecanismos de acción, entre los que se incluyen la infección activa por contacto con la cuticula, la ingestión de propágulos fúngicos por parte de las larvas, el ingreso a través de aberturas naturales al interior del huésped o la entomotoxicidad causada por metabolitos ^12^^,^^13^.

Además, varios autores sugieren la implicación de procesos enzimáticos que, sumados a la producción de metabolitos, son considerados como los principales mecanismos de acción causantes de la mortalidad de las larvas de *Ae. aegypti*^15^^,^^36^^-^^38^.

Por otro lado, el incremento en la mortalidad y un menor tiempo requerido en el proceso, han sido ampliamente relacionados con el aumento en la concentración del inóculo aplicado ^35^^,^^39^. En este caso, aun cuando la concentración de conidios necesaria para matar al 50 % de una población larval de mosquitos (CL_50_) fue mayor que la publicada en otros reportes, tanto para el caso de *Trichoderma* sp. como para otros hongos biocontroladores ^14^^,^^39^^,^^40^, los autores de este estudio consideran que el uso de la cepa AP-91 seria de interés como herramienta de control vectorial. Esto también tiene como soporte el TL_50_ reportado, ya que las 21 horas de exposición, requeridas para la eliminación del 50 % de una población larval de *Ae. aegypti,* resulta adecuado en función del tiempo de permanencia del mosquito en el ambiente acuático.

El TL_50_ obtenido (20,67 horas para AP-91) fue menor que el reportado por otros autores con otras especies de hongos. Tal es el caso de un estudio en donde se utilizaron varias cepas de *Metarhizium anisopliae* -nativas de Buenos Aires (Argentina)- en larvas de *Ae. aegypti,* en donde se encontró que el TL_50_ variaba entre seis y siete dias, usando una concentración de 5 * 10^7^ conidios/ml, ligeramente inferior a la utilizada en este estudio ^41^. Asimismo, existen reportes de *Beauveria bassiana* con un TL_50_ de 48 horas, en el mismo tipo de larvas y con una concentración de 1 * 10^12^ conidios/ ml, mucho mayor que la aplicada en los bioensayos aqui reseñados ^42^. Dado que el TL_50_ registrado en el presente estudio fue de 21 horas aproximadamente, podria considerarse adecuado para que esta cepa ejerza un efecto de biocontrol.

Este trabajo es de gran importancia por describir el hallazgo de la cepa AP-91 de *Trichoderma* sp. en la región del Urabá, una zona endémica para el dengue, y lograr su aislamiento a partir de larvas centinelas de *Ae. aegypti*^43^^,^^44^. La cepa en estudio presentó gran patogenicidad, por lo que podría convertirse en una candidata clave para controlar el mosquito en áreas donde las arbovirosis son un problema critico de salud pública. Su eficacia en condiciones de campo o semicampo, asi como su inocuidad frente a otros organismos no blanco, serán abordados en investigaciones futuras.
